# Right ventricular shape and function: cardiovascular magnetic resonance reference morphology and biventricular risk factor morphometrics in UK Biobank

**DOI:** 10.1186/s12968-019-0551-6

**Published:** 2019-07-18

**Authors:** Charlène Mauger, Kathleen Gilbert, Aaron M. Lee, Mihir M. Sanghvi, Nay Aung, Kenneth Fung, Valentina Carapella, Stefan K. Piechnik, Stefan Neubauer, Steffen E. Petersen, Avan Suinesiaputra, Alistair A. Young

**Affiliations:** 1 Department of Anatomy and Medical Imaging, University of Auckland, Auckland, New Zealand; 2 Auckland Bioengineering Institute, University of Auckland, Auckland, New Zealand; 3 William Harvey Research Institute, NIHR Barts Biomedical Research Centre, Queen Mary University of London, Charterhouse Square, London, UK; 4 Oxford NIHR Biomedical Research Centre, Division of Cardiovascular Medicine, Radcliffe Department of Medicine, University of Oxford, Oxford, UK; 5 Department of Biomedical Engineering, King’s College London, London, UK

**Keywords:** Cardiovascular magnetic resonance, Ventricular function, Atlases, UK biobank

## Abstract

**Background:**

The associations between cardiovascular disease (CVD) risk factors and the biventricular geometry of the right ventricle (RV) and left ventricle (LV) have been difficult to assess, due to subtle and complex shape changes. We sought to quantify reference RV morphology as well as biventricular variations associated with common cardiovascular risk factors.

**Methods:**

A biventricular shape atlas was automatically constructed using contours and landmarks from 4329 UK Biobank cardiovascular magnetic resonance (CMR) studies. A subdivision surface geometric mesh was customized to the contours using a diffeomorphic registration algorithm, with automatic correction of slice shifts due to differences in breath-hold position. A reference sub-cohort was identified consisting of 630 participants with no CVD risk factors. Morphometric scores were computed using linear regression to quantify shape variations associated with four risk factors (high cholesterol, high blood pressure, obesity and smoking) and three disease factors (diabetes, previous myocardial infarction and angina).

**Results:**

The atlas construction led to an accurate representation of 3D shapes at end-diastole and end-systole, with acceptable fitting errors between surfaces and contours (average error less than 1.5 mm). Atlas shape features had stronger associations than traditional mass and volume measures for all factors (*p* < 0.005 for each). High blood pressure was associated with outward displacement of the LV free walls, but inward displacement of the RV free wall and thickening of the septum. Smoking was associated with a rounder RV with inward displacement of the RV free wall and increased relative wall thickness.

**Conclusion:**

Morphometric relationships between biventricular shape and cardiovascular risk factors in a large cohort show complex interactions between RV and LV morphology. These can be quantified by z-scores, which can be used to study the morphological correlates of disease.

**Electronic supplementary material:**

The online version of this article (10.1186/s12968-019-0551-6) contains supplementary material, which is available to authorized users.

## Background

Large epidemiological studies have established hypertension, smoking, diabetes, and hyperlipidaemia as factors associated with increased risk of adverse cardiovascular events [1–3]. Heart shape and function also adapt in response to subclinical disease processes, and study of the effects of risk factors on heart shape and function can give insight into the mechanistic processes leading to symptomatic disease. Although relationships between global ventricular mass and volume have been well studied [4–6], associations with regional shape and motion remain poorly understood. In particular, interactions between right ventricular (RV) and left ventricular (LV) shape and function [7, 8] imply the need to study both in combination, as changes in one ventricle are likely to affect the other.

Atlas-based analysis can provide detailed information on shape variations and their relationships with disease processes. These methods have enabled robust and precise quantification of relationships between LV shape and risk factors in the asymptomatic population [9, 10], and remodeling after myocardial infarction [11]. LV chamber dilatation, sphericalization and hypertrophy have also been associated with obesity [12] and hypertension [13]. However, changes in RV geometry and function are also important in many cardiovascular diseases [14–17] but are less well understood due to the complex shape of the chamber.

The goal of this study was to establish a reference healthy morphology, and investigate the RV variations and biventricular interactions associated with common risk factors using a biventricular atlas. We developed an automated framework to customize a 3D subdivision surface mesh to contour and landmark positions manually defined at end-diastole (ED) and end-systole (ES) in over 4000 participants of the UK Biobank cardiovascular magnetic resonance (CMR) imaging extension. The atlas captured the shape and motion of both LV and RV as well as the mitral and tricuspid valves’ positions and orientations. The strength of associations between biventricular shape and cardiovascular risk factors and disease were evaluated by computing morphological scores using logistic regression analysis. This enabled characterization of the RV variation as well as biventricular interactions associated with each risk factor.

## Methods

### Study population

We assessed CMR examinations and data from the first 4989 participants obtained from the UK Biobank. The UK Biobank study is a large prospective study designed to identify the causes of complex diseases by collecting questionnaire data, physical measurements and biological samples from 500,000 individuals in the UK [18]. In 2015, the UK Biobank extended their study into imaging, including brain, heart, whole body composition, carotid artery, bone and joint imaging, with the aim to scan 100,000 participants by 2023. All participants gave written informed consent and the appropriate institutional review boards approved the study protocol (National Research Ethics Service North West 11/NW/0382).

### CMR imaging protocol and image analysis

The UKB CMR protocol has been described previously [19]. Briefly, all imaging was performed on a wide bore 1.5 T scanner (MAGNETOM Aera, *Syngo* Platform VD13A, Siemens Healthineers, Erlangen, Germany) using a phased-array cardiac coil. Retrospectively gated balanced steady state free precession cine images were acquired with three long axis orientations (horizontal long axis, vertical long axis, LV outflow tract) and a complete short axis stack covering both the RV and the LV (6 mm thickness for long axis, 8 mm thickness for short axis, flip angle 80°, TR/TE = 2.6.1.1 ms, temporal resolution 32 ms interpolated to 50 phases per cardiac cycle, pixel size 1.8 × 1.8 mm). Each slice was acquired in a separate breath-hold.

Contours and landmarks were defined manually by eight observers independently across two core-laboratories using cvi^42^ post-processing software (Version 5.1.1, Circle Cardiovascular Imaging Inc., Calgary, Canada) [20]. Inter-observer variabilities have been reported previously [20] with intra-class correlation coefficient 0.97 for LV end-diastolic volume (EDV), 0.88 for LV end-systolic volume (ESV), 0.92 for RVEDV and 0.77 for RVESV. RV and LV endocardial borders, LV epicardial borders, and left and right atrial endocardial borders were drawn at both ED and ES in accordance with the Society for Cardiovascular Magnetic Resonance recommendations [21]. The ED frame was selected as the first frame after detection of the R wave, and the ES frame was selected as the smallest LV blood pool area in the mid-ventricular slice. At both ED and ES, the most basal slice had at least 50% of the LV blood pool surrounded by myocardium. LV papillary muscles were excluded from the LV mass but included as part of the LV ED volume to reduce inter-observer variability. Tricuspid and mitral valves points were defined from left atrial contours delineated on the two-chamber and four-chamber long axis images, and right atrial contours on the four-chamber long axis images.

### Cardiovascular disease risk factors and reference healthy group

Four preclinical cardiovascular disease (CVD) risk factors: high cholesterol, smoking, high blood pressure, and obesity, were selected for the analysis based on previous studies [6]. Three disease factors, presence of diabetes, previous angina and previous myocardial infarction (MI), were also examined. All factors were categorised as dichotomous variables. Smoking status was defined as self-reported current smoker vs non-smoker at the time of CMR examination. Diabetes and high cholesterol were determined by participants’ answers to binary questionnaire items: e.g. diabetes diagnosed by a doctor, diagnosis of high cholesterol, or use of medication for cholesterol. Obesity was defined as body mass index (BMI) more than 30 kg/m^2^. Previous MI or angina were determined by participant self-reporting, including previous diagnosis by a doctor or current use of medications.

In [20], a sub-cohort from the same UK Biobank participants was determined by excluding participants > 74 years, known CVD or symptoms of disease, and other conditions or medications relating to conditions known to affect cardiac chamber size and function. Due to the small number of non-Caucasian participants, these were also excluded. In this study, we applied the same criteria, and also excluded any participants with high blood pressure or any of the risk factors and diseases defined above as recorded on the same visit as the CMR scan, to define the reference healthy group. Further details of exclusion criteria are given in Petersen et al. [20].

Cases without adequate information on risk factors, or adequate RV endocardial contours, valve points or apical points to form a biventricular geometry, were excluded from our study, leaving 4329 cases included in the atlas. After all exclusions, 630 cases were included in the reference group. Characteristics of the participants included, and the reference group, are shown in Table 1.

**Table 1:** Characteristics of the participants included in the atlas, and the reference healthy sub-cohort. The data are presented as mean ± std. dev. or number of positives (% of total). MI, myocardial infarction

	Total	Reference healthy sub-cohort
n	4329	630
Age (years)	62.0 ± 7.5	59.1 ± 6.3
Male	2057 (48%)	290 (46%)
Weight (kg)	75.8 ± 15.3	69.2 ± 11.7
Height (cm)	169.8 ± 9.4	169.9 ± 8.1
Smoking	280 (6%)	0
High cholesterol	294 (7%)	0
Previous MI	93 (2%)	0
Diabetes	223 (5%)	0
Angina	101 (2%)	0
High blood pressure	1125 (26%)	0
Obesity	709 (16%)	0

### Patient-specific biventricular surface mesh

**Fig. 1:**
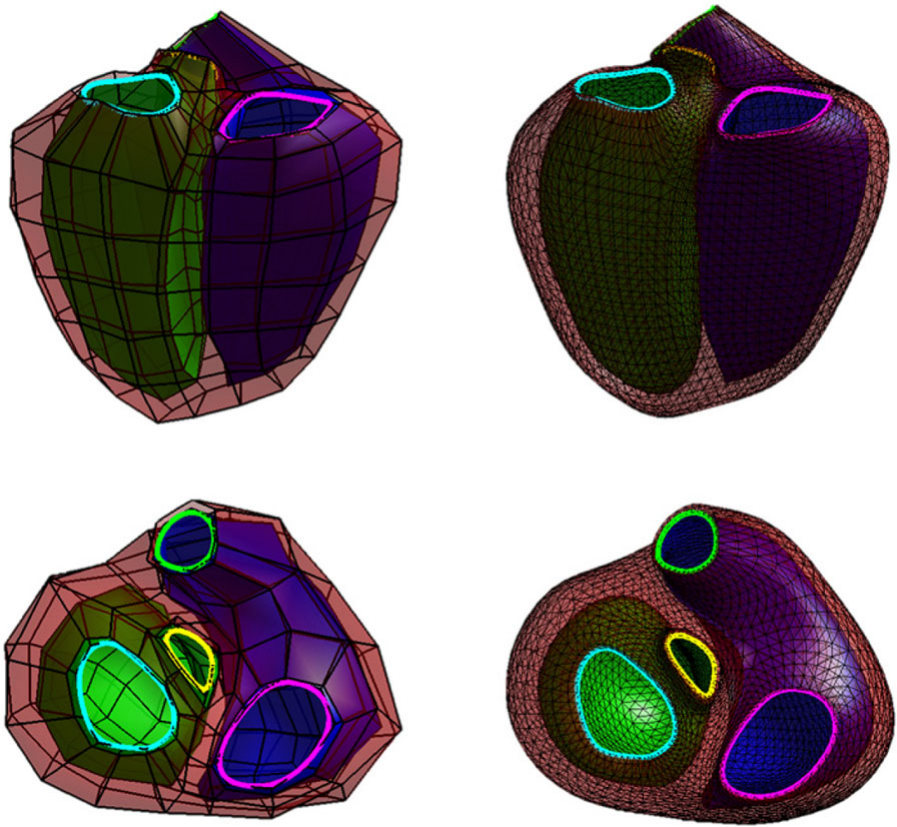
Biventricular coarse mesh (left) and its Catmull Clark subdivision at level 2 (right). The left ventricle (LV) is shown in green, the right ventricle (RV) in blue and the epicardium in red. The mitral valve is highlighted in cyan, the aortic valve in yellow, the tricuspid valve in magenta and the pulmonary valve in green

A biventricular subdivision surface template mesh was constructed as described previously in [22]. This included the LV, RV, and the four valves (aortic, mitral, tricuspid and pulmonary). The coarse mesh comprised a total of 182 hexahedral elements with 388 vertices. To generate a smooth surface mesh, this coarse mesh was then subdivided twice using the Catmull-Clark algorithm [23, 24]. Figure 1 shows the template coarse mesh (top row) and the final smooth surface mesh (bottom row). The final surface mesh consisted of 5352 unique vertices. During the subdivision process, sharp edges [25] were maintained at the RV insertions and the four valves.

The epicardium of the RV free wall was not manually contoured, however the biventricular epicardium was required to customize the surface mesh. Therefore, we estimated the RV epicardial mesh points by extending the RV endocardium mesh points by a fixed distance perpendicular to the endocardial surface, determined by an average RV wall thickness for ED (3 mm) and ES (5 mm) [26]. Note that the RV epicardium was only used to complete the biventricular model during the fitting, but was not included in subsequent analyses.

**Fig. 2:**
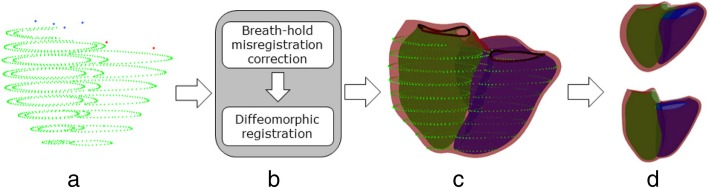
Atlas construction pipeline: **a** 3D contour points extracted from 2D contour points. Mitral points are shown in blue and tricuspid points in red. **b** Registration framework. **c** Registration results. **d** Principal component analysis of shape variation. Upper and lower panel show ±2 std. dev in the fourth mode at end-diastole (ED)

Figure 2 shows the automated biventricular atlas construction pipeline, where contours were the input. Patient-specific biventricular mesh customization was performed in two steps: 1) correction of breath-hold misregistration between short-axis slices, and 2) deformation of the subdivided template mesh to fit the manual contours while preserving the topology of the mesh. To correct breath-hold misregistration, we adapted the method described previously in [9]. Briefly, a stiff linear least squares optimization was performed only on the LV with the long-axis images using the D-Affine regularization method [27]. Next, 2D contour intersections between the surfaces and short axis slices were generated, and the centre of each contour was calculated by using the area-weighted average of the barycentre defined by the area of the intersected contour. The longitudinal line that aligns with these centres was determined as the new centre to shift the short axis images so that the contour barycentre aligned with the new centre. Contours were shifted in two directions within the image plane (in-plane). This breath-hold misregistration correction process was applied to all short-axis slices.

After correcting for the slice misregistration, the coarse mesh was customized to each case using a diffeomorphic registration algorithm that deforms the surfaces while preserving the topology of the heart [22]. Briefly, the algorithm consists of two iterative processes: 1) a linear least-squares optimization, which provides an initial solution close to the contours, 2) a non-linear optimization process with explicit diffeomorphic constraints to avoid epicardial and endocardial surface intersection.

Two quality metrics were used to assess the quality of a patient-specific biventricular mesh: 1) global point-to-surface distances between the manual contour points and the customized mesh, and 2) cardiac volume comparisons between the customized mesh and the manual contours, including EDV, ESV, stroke volume (SV), and ejection fraction (EF) of both ventricles. The first metric gives information on the surface errors and the second metric quantifies volume errors using the standard slice summation method as a reference.

### Biventricular atlas

To build the biventricular atlas, all the ED mesh points were first aligned by a rigid registration of translation and rotation using the Procrustes algorithm. This transformation was then applied to the ES mesh. ED and ES surface points were then concatenated to form a long vector. Principal component analysis (PCA) was subsequently performed on the NxP matrix, where *N* = 4329 is the number of subjects and *P* = 32,112 is the number of ED and ES 3D (x,y,z) vertex coordinates. PCA computes the principal components of shape variation across the cohort, ordered by amount of variance explained [28]. Using a concatenation of ED and ES shapes instead of ED or ES alone captures the variation in function (motion between ED and ES) as well as shape in the statistical atlas. Pulmonary and aortic valve points, and RV free wall epicardial points were removed from the surface meshes prior to PCA, since data on these areas were not available in the manual contours.

### Strength of geometric relationships

In order to quantify the strength of the relationships between the biventricular shapes and the risk factors, we constructed regression models using penalised logistic regression with elastic net regularization [29]. Briefly, this method optimizes the logistic function and penalizes both the absolute value of the coefficients, in order to shrink irrelevant coefficients to zero, and the squared size of the coefficients, to limit the impact of collinearity.

For each risk factor, a separate regression model was built using that factor as the univariate dependent variable, and the PCA component scores as the independent variables. Participants with positive coding for the risk factor were selected as positives, and participants in the reference healthy group were selected as negatives. Any participants who also had previous MI and angina were removed from the positives. For angina, only participants who were positive for previous MI were removed. For previous MI, no participants were removed.

Receiver operating characteristic (ROC) and precision recall (PR) curves were generated to quantify the strength of relationships in the presence of imbalanced classes (number of positives being typically less than the number of negatives) [30, 31]. An area under the ROC curve (AUC) of 0.5 suggested no discrimination (50% chance of distinguishing positive from negative cases), 0.7 to 0.8 was acceptable, 0.8–0.9 was excellent and > 0.9 was outstanding [32]. To calculate optimal performance, a cut-off value was calculated using the Youden index [33], maximizing both specificity and sensitivity values.

The above shape regression models were compared with a baseline model constructed using LVEDV, RVEDV, LVESV, RVESV, and LV mass (LVM) as independent variables. For these comparisons the covariates of age, sex, height and weight were not included as independent variables as they obscured the strength of associations with geometric factors alone. As above, a ten-fold cross-validation was performed. For each fold, the average ROC (aROC) and average PR (aPR) for the test sets across the ten folds were calculated for each risk factor and the average specificity, sensitivity (or recall), precision (or positive predictive value), F1-score, AUC and cut-off values were calculated.

### Morphometric associations with risk factors

Associations between the biventricular shape and risk factors were examined further using multiple linear regression. In this multivariate model, the PCA component scores were treated as the dependent variables and the independent variables comprised age, sex, height and presence of the risk and disease factors. A morphometric mode was estimated for each risk factor and disease variable, by partitioning the contribution of each factor to the shape. From this morphometric mode, a score can be automatically calculated and standardized to produce morphometric scores. The visualizations and scores are therefore corrected for confounding factors such as age, sex and height, as well as the presence of multiple risk factors and the presence of disease (see Appendix for details).

In addition to the morphometric scores and shape modes, traditional measures such as volume and dimensions could be calculated from the morphometric shapes for each factor. These included EDV, ESV, EF, LVM, RV longitudinal shortening, RV eccentricity, RV sphericity, LV relative wall thickness, and LV mass-to-volume ratio. RV longitudinal shortening was computed as the change in length from the middle of the tricuspid valve to the RV apex between ED and ES divided by the length at ED. RV sphericity was defined as the ratio between the base-apex length and the perpendicular distance between the RV free wall and the septum. RV eccentricity was calculated as the ratio of the minor axis parallel to the septum divided by the minor axis perpendicular to the septum [34]. The eccentricity index was calculated as the ratio of the LV minor axis parallel to the septum divided by the LV minor axis perpendicular to the septum [35]. LV sphericity was calculated as the EDV divided by the volume of a sphere with a diameter corresponding to the LV major axis at ED in the long axis four chamber view [36]. The LV relative wall thickness was defined as twice the posterior wall thickness divided by the ED diameter.

### Statistical analysis

Statistical evaluation was carried with R [37] and the *caret* package (http://caret.r-forge.r-project.org/). ROC and PR curves were generated using the *pROC* package [38]. Significant differences between ROC curves were calculated using DeLong’s test on the average ROC, available from the *pROC* package. Two-tailed paired t-tests of cardiac volumes measured between the automated methods with manual contours were performed. *P* values of < 0.005 were considered as statistically significant.

## Results

### Patient-specific biventricular mesh

**Fig. 3:**
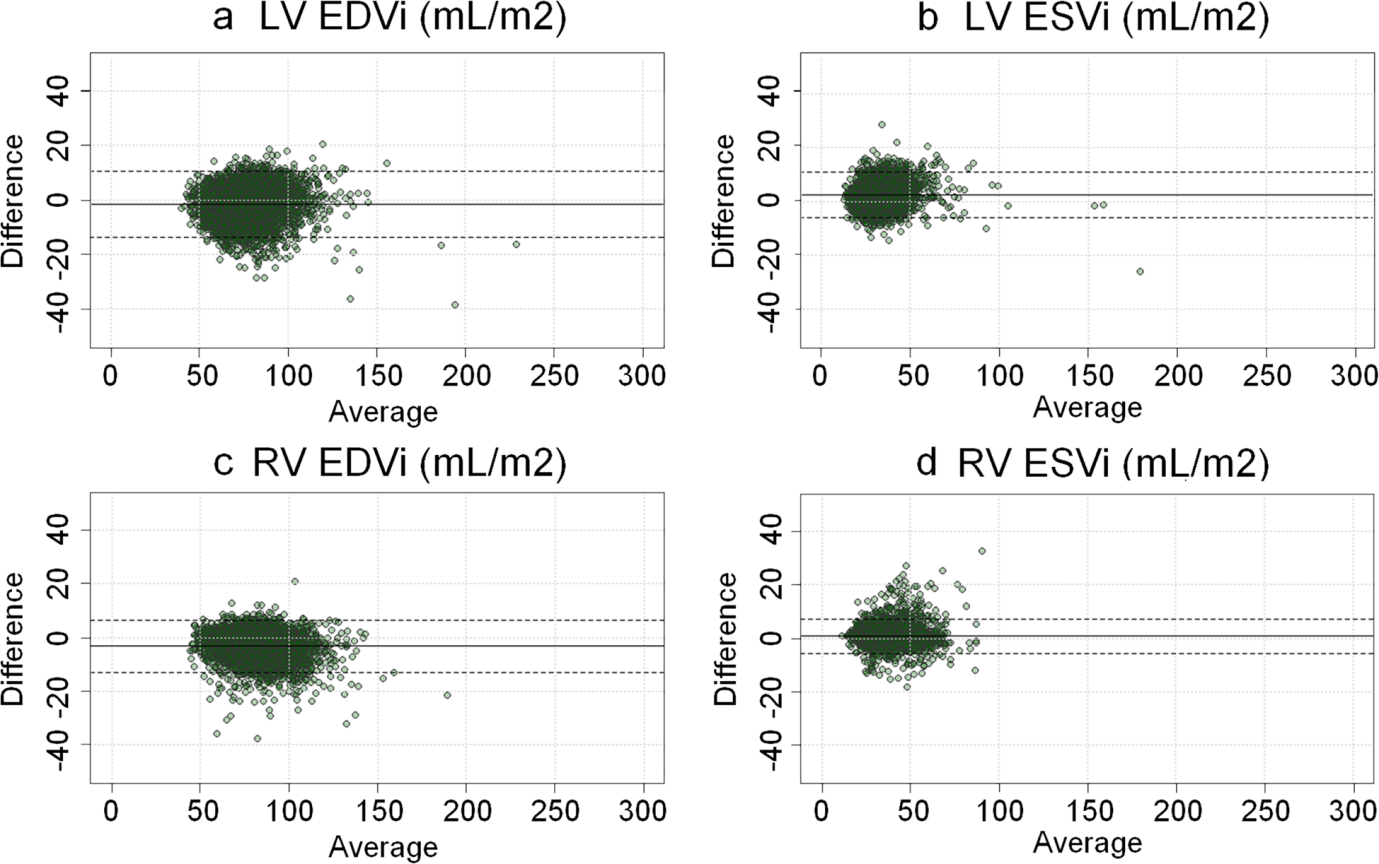
Bland-Altman agreement plots between biventricular mesh fitting and manual analysis. **a** LV end-diastolic volume index, **b** LV end-systolic index, **c** RV end-diastolic index, and **d** RV end-systolic index. Mean biases and ± 2 std. dev of measurement differences are shown as horizontal dashed lines. LV = left ventricle. RV = right ventricle

The biventricular mesh template was successfully customized to all cases using the fully automatic process (Fig. 2). RV and LV volumes calculated from the mesh using numerical integration agreed well with the volume computed from manual contours using standard slice summation. RV EDV and ESV biases were and − 6.0 ± 9.4 ml (*p* < 0.005) and 1.6 ± 6.3 ml (*p* < 0.005) respectively. LVEDV and ESV biases were − 2.6 ± 11.3 ml (*p* < 0.005) and − 5.2 ± 8.2 ml (*p* < 0.005) respectively. Figure 3 shows the Bland-Altman agreement plots of the LV and RV volumes between the biventricular mesh with manual contours. The average point-to-surface distances between the contour points and the customized mesh surfaces were less than 2 mm. For the RV endocardium, the distances were 1.5 ± 0.9 mm, for the LV endocardium were 1.2 ± 0.7 mm and for the epicardium were 1.3 ± 0.8 mm. The average in-plane translation for breath-hold misregistration correction was 2.8 ± 1.5 mm, consistent with previous results [9, 39].

### Atlas principal components

**Fig. 4:**
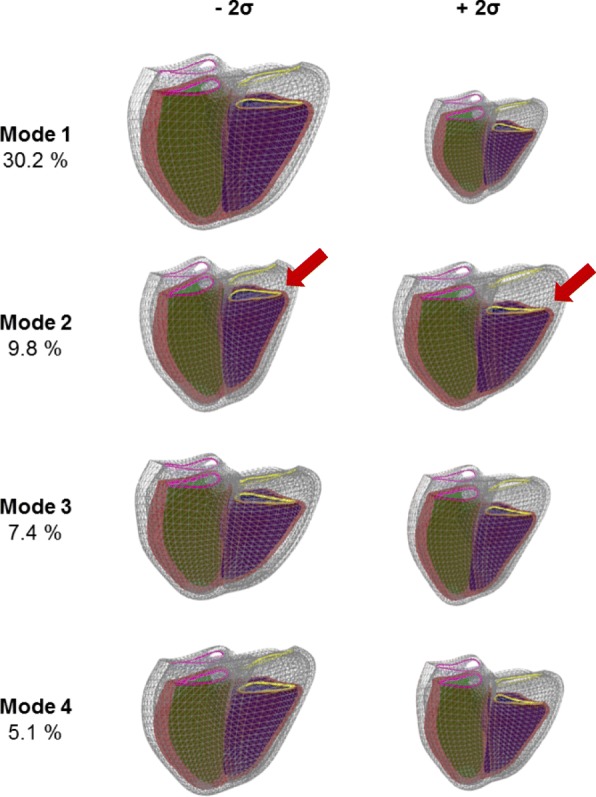
First four components of variation for the combined end-diastole (ED) and end-systole (ES) atlas. The ED is shown in wireframe and the ES in solid mesh. The right ventricle is shown in blue, the LV in green and the LV epicardium in red. The mitral valve is shown in pink and the tricuspid valve in yellow. The red arrow shows changes in the motion of the tricuspid valve. The left and right shapes of each mode correspond to the mean ± 2 std. dev. Animations of these shape components have been provided in the Additional file 1: Figure S2

**Fig. 5:**
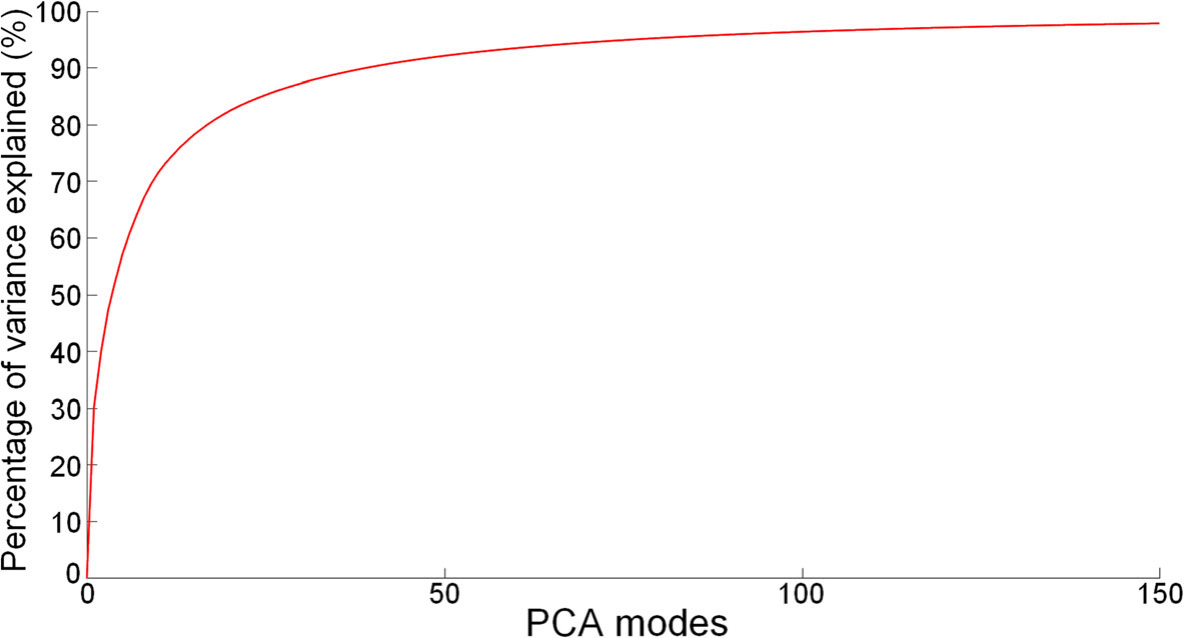
Percentage of variance explained as a function of mode for the atlas

Figure 4 visualizes the first four principal components of the biventricular shape variations by showing the shapes at ±2 standard deviation from the average shape. Animations of these components can be seen in the Additional file 1: Figure S2. These first four modes represent the biggest variations of the LV and RV shapes across the 4329 cases. Additionally, since ED and ES shapes were concatenated in the PCA calculation, Fig. 4 also visualizes variations in motion between ED and ES frames. The biggest variation with 30.2% (Mode 1) was associated with the size of the heart. The second mode (9.8%) shows differences in the motion of the tricuspid valve (red arrow), which can be associated with the tricuspid annular plane systolic excursion (TAPSE). The third (7.4%) and fourth (5.1%) modes were visually associated with the height to width ratio (sphericity) of the ventricles, and the base orientation, respectively. The first 50 PCA modes accounted for 92.1% of the total shape variance. Figure 5 shows the percentage of variance explained as a function of mode number.

### Strength of geometric relationships

**Fig. 6:**
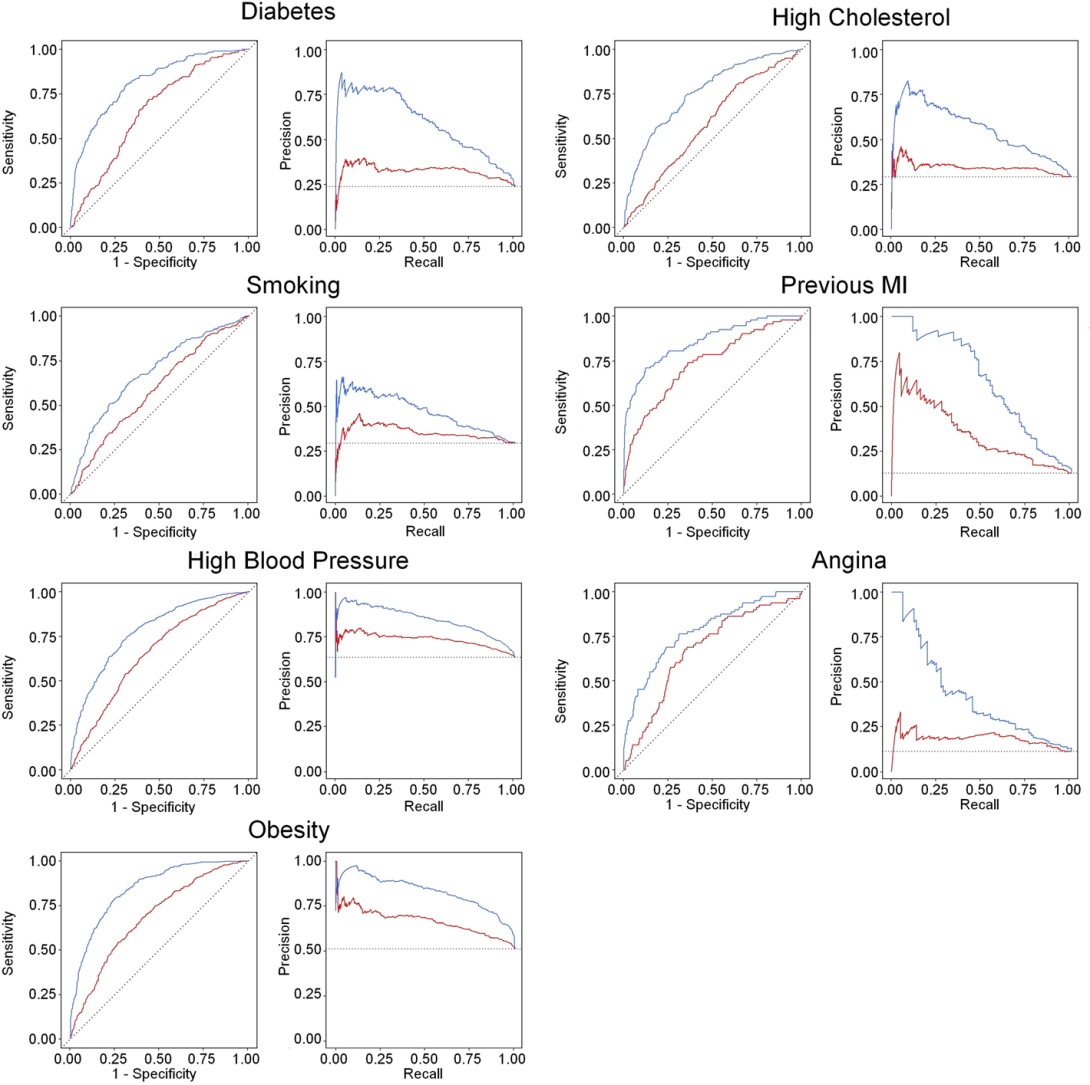
Receiver Operating Characteristics (ROC) curves and Precision-Recall curves for each risk factor regression model. MassVol and PCA50 models are shown by red and blue curves, respectively

Table 2 shows the results of the cross-validation logistic regression models for the shape atlas, comparing a model using the first 50 PCA modes (PCA50), with a baseline model using standard mass and volumes as independent variables (MassVol). In general, the PCA50 models showed higher sensitivity (recall), specificity, precision (positive predictive value), F1-score (a harmonic average of precision and recall) and negative predictive value at the Youden cutoff. Using the DeLong test, the PCA50 models showed significantly higher AUC than the standard MassVol model for previous MI, high cholesterol, smoking, diabetes, obesity, angina, and high blood pressure. ROC and PR curves are shown in Fig. 6. All PCA50 PR curves were above the corresponding MassVol curves, showing a higher precision for each value of recall than the MassVol models. For the PCA50, angina, smoking, high blood pressure, and high cholesterol regression models showed an acceptable AUC. Presence of diabetes, previous MI and obesity showed an excellent AUC. For MassVol, only the previous MI regression model showed an acceptable AUC.

**Table 2:** Ten-fold cross-validation results from penalized logistic regression models using the first 50 PCA modes (PCA50) and from the reference baseline mass/volumes (MassVol) measurement models

Factor	Model	AUC	Sensitivity	Specificity	Precision	F1-score	npv	Cutoff
Cholesterol	MassVol	0.587	0.704	0.451	0.346	0.464	0.787	0.495
	PCA50*	0.751	0.792	0.579	0.437	0.564	0.871	0.485
Smoking	MassVol	0.594	0.856	0.287	0.334	0.480	0.826	0.490
	PCA50*	0.699	0.525	0.795	0.517	0.521	0.800	0.511
Diabetes	MassVol	0.652	0.740	0.554	0.342	0.468	0.872	0.494
	PCA50*	0.817	0.704	0.781	0.502	0.586	0.894	0.504
Previous MI	MassVol	0.733	0.699	0.663	0.235	0.351	0.937	0.490
	PCA50*	0.853	0.677	0.911	0.529	0.594	0.950	0.518
Angina	MassVol	0.671	0.700	0.637	0.196	0.307	0.944	0.497
	PCA50*	0.773	0.663	0.763	0.262	0.376	0.947	0.493
Obesity	MassVol	0.682	0.675	0.598	0.638	0.656	0.637	0.494
	PCA50*	0.839	0.760	0.763	0.771	0.766	0.752	0.500
Hypertension	MassVol	0.651	0.693	0.543	0.718	0.705	0.514	0.495
	PCA50*	0.770	0.752	0.663	0.789	0.770	0.615	0.494

Statistical tests were performed between MassVol and PCA50. Significant differences (*p* < 0.005) are represented with an asterisk

*PCA* principal component analysis, *MI* myocardial infarction

### Morphometric associations with risk factors

**Fig. 7:**
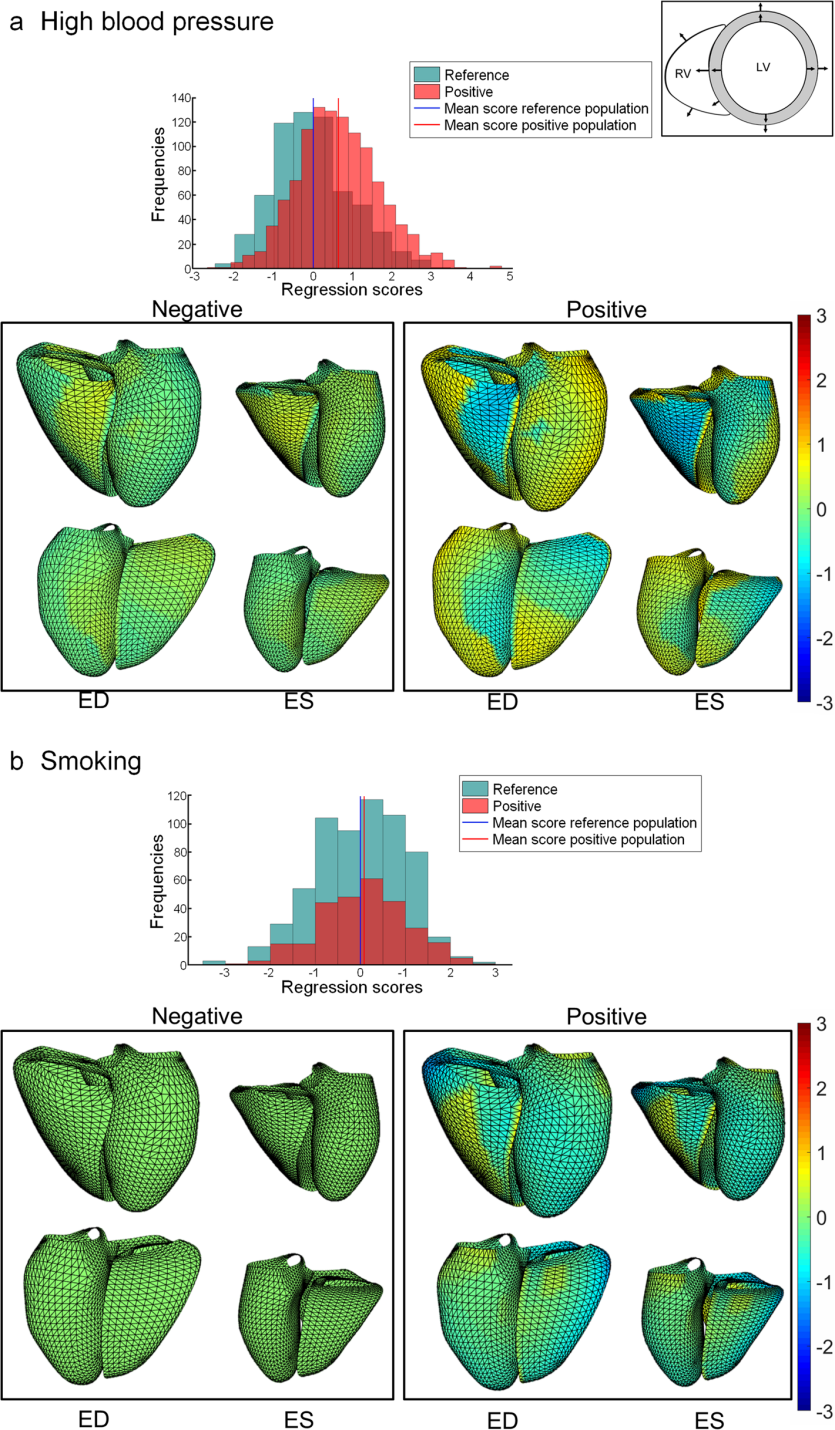
Shape changes due to high blood pressure (**a**) and smoking (**b**), adjusted for age, sex, height and other factors. Colors denote difference from the mean shape in mm (red outward, blue inward) for factor positive (Positive) and factor negative (Negative) groups. Outward directions are shown on the top right picture. Top row: anterior view of the RV (left) and LV (right). Bottom row: posterior view (left: LV, right: RV). Histograms show morphometric scores for reference healthy group and factor positive group

Morphometric scores and shape modes were calculated using a multivariate regression model with the first 50 PCA modes as dependent variables, and age, sex, height, risk factors and disease factors as independent variables, as detailed in the Appendix. Significant effects were found for several risk factors for specific principal components. The first principal component (associated with size) was significantly increased with high blood pressure, obesity, and previous MI and decreased in diabetes and high cholesterol. The second principal component (associated with TAPSE) was significantly decreased with smoking, high blood pressure and obesity as well as diabetes, previous MI and angina. The third principal component (associated with sphericity) was significantly increased with obesity and previous MI. Figure 7 shows average morphometric shape changes associated with high blood pressure (Fig. 7a) and smoking (Fig. 7b). Red colours refer to change of position outward from the LV or RV free wall, and for the septum outward from the LV (inward to the RV), relative to the mean. High blood pressure was associated with an outward displacement of the LV free wall and an inward displacement of the LV side of the septum at ES, together with an outward displacement of the RV side of the septum (towards the RV) resulting in a thickening of the septum and contributing to the decreased RVESV. For smoking, regional shape changes were found in both RV and LV. The septum shifted rightwards at ES while most of the free walls moved inward, except for regions around the valves. Visualizations of the changes associated with the other risk factors can be found in the online Additional file 2: Figure S1. As a comparison, changes associated with age and sex (Additional file 2: Figure S1) show that males had larger ventricles and older people had relatively smaller ventricles, as found previously [6, 40, 41].

Table 3 shows how the volumes and dimensions derived from average shapes in each class change with each risk or disease factor. Percentage change corresponds to the difference between the mean shape for the negatives and the mean shape for the positives for each factor. Smoking was associated with shapes with lower volumes, higher RV eccentricity and lower RV shortening, and higher LV mass to volume ratio and relative wall thickness. High cholesterol was associated with lower volumes and mass, smaller RV shortening and higher LV sphericity. High blood pressure was associated with smaller RV and greater LV volumes, higher RV shortening and smaller RV eccentricity and sphericity, along with higher LV mass to volume ratio and relative wall thickness. Obesity was associated with greater RV and LV volumes, greater RV sphericity and smaller RV shortening and eccentricity, and increased LV mass to volume ratio and relative wall thickness. Diabetes was associated with lower RV and LV volumes, but higher LV mass to volume ratio. Previous MI was associated with increased RV and LV volumes, increased RV shortening and RV eccentricity, decreased LV mass to volume ratio, and decreased LV shortening.

**Table 3:** Volume and dimension differences (%) between the positive and negative means generated from each of the linear regression morphometric modes

	Smoking	Chol	Previous MI	Diabetes	Angina	HBP	Obesity
RV EDV	−3.1	−1.5	4.2	−3.4	0.7	−0.1	8.5
RV ESV	−1.7	−2.1	4.3	−2.6	−1.1	−2.4	10.1
RV Eccen.	0.514	−0.77	3.91	−0.58	− 0.38	− 0.89	− 0.72
RV Spher.	− 0.08	1.2	0.04	− 0.24	0.77	−0.33	1.21
RV Short.	−1.74	− 1.8	2.19	−0.02	1.85	1.84	−1.36
LV EDV	−1.7	−2.2	11.7	−3.3	1.6	1.4	7.3
LV ESV	0.6	−4.2	18.4	−2.5	0.8	1.0	7.8
LV Mass	−0.5	−3.1	5.4	−1.3	1.7	5.1	13.9
LV MVR	1.2	−0.8	−7.1	1.9	0.1	3.7	7.1
LV RWT	1.1	−0.7	−5.8	1.2	0.1	3.1	6.4
LV Eccen.	−0.07	0.2	0.02	0.6	0.1	0.9	1.4
LV Spher.	−0.2	−0.2	8.4	0.1	1.0	−1	2.1
LV Short.	−0.9	0.8	−3.6	0.2	1.8	0.8	−0.1

*EDV* end-diastolic volume, *ESV* end-systolic volume, *Eccen*. eccentricity, *Spher*. sphericity, *Short* longitudinal shortening, *LV MVR* left ventricular mass to volume ratio, *LV RWT* left ventricular relative wall thickness

## Discussion

There are limited data on the morphological changes associated with risk factors in the general population. This study provided a direct comparison of relationships between RV and LV geometry and motion and common risk factors and disease. Analysis of both ventricles is motivated by the anatomical coupling of a continuous system of muscle fibers, a common septal wall, and enclosure within the pericardium [42]. Both acute and chronic interactions have been observed previously [43]. Here, we found that shape scores had stronger relationships with risk factors and disease than traditional mass and volume measures. The contributions of each factor, corrected for the presence of other factors and age, sex, and height, could be quantified, visualized and expressed as a morphometric score. The atlas shape features incorporated both ED and ES shape and the motion between, with many factors associated with changes in contraction patterns.

In this study, the principal component explaining the most variation of biventricular shape was associated with the size, as found in previous studies [9, 44]. We found significant increases in size due to high blood pressure and previous MI but decreases due to diabetes and cholesterol. The decrease in size found in patients with high cholesterol may be due to medication use since statins are known to reduce heart size. The second mode was associated with the tricuspid valve motion. The finding that this mode accounted for the most shape variation after heart size is particularly striking since previous atlases constructed using only LV contours found that sphericity was the second mode after size, not valve excursion [10]. Previous CMR RV atlases [44–46] did not show this variation as mitral and tricuspid valve points were not included. TAPSE is known to be a sensitive index with prognostic value [47]. We found reductions in longitudinal shortening with smoking, and obesity as well as diabetes. The third mode was associated with width to height ratio (sphericity) of both ventricles. Previous LV atlases found sphericity to be an important shape feature [9, 10]. Sphericity is known to be associated with adverse remodelling process in asymptomatic populations and patient with CVD [48]. In our study, increases in RV sphericity mode were found with obesity and angina.

The results of this study are in accordance with previous studies of mass and volume changes [6, 13, 49] and extend these findings to the visualization of regional changes for common cardiovascular risk factors. In the Multi-Ethnic Study of Atherosclerosis (MESA), high blood pressure and obesity were associated with larger RV volume, and smoking and diabetes with reduced RV volume [4, 5]. This agrees with the changes in biventricular shape shown in Table 3. Males were also found to have larger RV volumes than females (corrected for body habitus) and a cohort effect of reduced RV volume in older participants [40], both of which were seen in the morphometric modes (Additional file 2: Figure S1). In the UK Biobank, obesity and high blood pressure were associated with increased RV volumes, whereas presence of diabetes or high cholesterol resulted in smaller volumes [6], in agreement with Table 3. The finding of increased RV longitudinal shortening after previous MI in the face of decreased LV shortening may reflect RV compensation to counter the decreased LV function [50], or increased RV preload [51].

The morphometric scores may therefore have prognostic significance. In the LV, geometrical variations including wall thickness and sphericity have been shown to be determinants of functional mitral regurgitation after myocardial infarction [52] and decreased survival in heart failure [53]. Both excessively high and low sphericity were associated with adverse outcomes in MESA [49]. In the RV, hypertrophy was associated with higher risk of heart failure or death in MESA [14]. In pulmonary hypertension, LV increased eccentricity, or roundness of the ventricle, was found to be predictive of adverse remodelling [15].

The availability of large scale CMR datasets has facilitated the utility of cardiac atlases. Bai et al. [44] used 1093 3D cine CMR studies of healthy subjects to build a 3D biventricular atlas. In a study of patients with severe pulmonary hypertension [17], RV statistical shape atlas features were used to predict survival with better performance than traditional mass and volume measures. McLeod et al. [54] have also found statistical shape variations related to arrhythmogenic right ventricular cardiomyopathy. Compared with our previous evaluation of LV atlases in a similar cohort [10], the biventricular analysis showed consistently higher AUC in Table 2, which suggests that incorporating the RV geometry improves the strengths of relationships with risk factors and disease.

One of the limitations of this study was the requirement for initial manual contouring. Machine learning methods are showing promise in registration and segmentation applications [55], and these will facilitate construction of highly reproducible shape models in the future. Another limitation of the study lies in the use of questionnaires and self-reporting of the participant. Also, neither blood cholesterol nor glucose data were available at this time and are therefore based on medication and self-reporting only in this study. It is well known that high cholesterol is associated with increased volumes and that statin medications decrease volumes [56]. Our findings might be therefore be confounded by medication use. With more cases becoming available in UK Biobank, it will be possible to examine the effects of each risk factor independently in more detail in the future. These would include evaluation of non-linear effects.

## Conclusions

In conclusion, RV shape and function could be quantified by atlas-based morphometric scores. Biventricular shape features had stronger associations with common risk factors than traditional measures of mass and volume. Shape changes associated with particular factors such as diabetes, affecting both the RV and LV, could be quantified using morphometric scores. These methods enable simple characterization of complex RV shape changes as well as biventricular interactions associated with pre-clinical disease processes. Each morphometric score can be calculated automatically as a single z-score value, which can be simply presented in a clinical report showing current status of a particular patient against a reference cohort, or longitudinal changes from previous exam. The biventricular atlas data used in this study is available from UK Biobank on request.

### Additional files

Additional file 1: **Figure S2.** Animations of the first four PCA components. (PPTX 2780 kb)

Additional file 2: **Figure S1.** Shape changes due to (a) previous MI, (b) angina, (c) obesity, (d) diabetes, (e) high cholesterol, (f) sex and (h) age, adjusted for other factors. Colors denote difference from the mean shape in mm (red outward, blue inward) for factor positive (Positive) and factor negative (Negative) groups. Outward directions are shown on the top right picture. Top row: anterior view of the RV (left) and LV (right). Bottom row: posterior view (left: LV, right: RV). Histograms show morphometric scores for reference healthy group and factor positive group. (PPTX 8496 kb)

## Data Availability

This research has been conducted using the UK Biobank resource under application 2964. The raw data, the derived data, the analysis and results have been clearly annotated and returned to UK Biobank, which will then incorporate the returned data into the central repository. UK Biobank will make the data available to all bona fide researchers for all types of health-related research that is in the public interest, without preferential or exclusive access for any person. All researchers will be subject to the same application process and approval criteria as specified by UK Biobank. For the detailed access procedure see http://www.ukbiobank.ac.uk/register-apply/.

## Appendix

The shape models were assembled into a data matrix X with N rows (cases) and P columns (3D surface points), and centered by subtracting the mean. The PCA shape modes Φ consisted of 50 columns each with P rows, comprising the principal component modes. The component scores were calculated by projecting shapes onto the modes as T = XΦ. A multivariate linear regression was used to estimate the contribution of each risk factor to the shape component scores, as follows.

$$ T=A\ B+R $$

where *T* are the PCA scores, *B* is the matrix of regression coefficients, and *R* is a matrix of residuals. *A* is the matrix of predictor variables, including age and height as continuous variables, and sex, high cholesterol, high blood pressure, obesity, smoking, diabetes, previous MI and angina as categorical variables. For the i^th^ variable in *A*, the outer product of the i^th^ column of *A* and the i^th^ row of *B* gives the contribution of that variable to the estimated shape scores:

$$ {T_i}^{est}={A}_i\otimes {B}_i $$

The corresponding shapes can be calculated as.

$$ {X_i}^{\mathrm{est}}={T_i}^{\mathrm{est}}\ {\varPhi}^T $$

The average predicted shapes for the positive and negative cases for that predictor variable can be calculated as *X*_*i*_^*+*^ and *X*_*i*_^*−*^ respectively. A morphometric mode can then be calculated as.

$$ {D}_i=\left({X_i}^{+}-{X_i}^{-}\right) $$

and normalized to have unit length. Morphometric scores can be calculated as.

$$ {S}_i={D_i}^TX $$

## Abbreviations

aPR: Average precision recall
aROC: Average receiver operator curve
AUC: Area under the curve
BMI: Body mass index
CMR: Cardiovascular magnetic resonance
CVD: Cardiovascular disease
ED: End-diastole
EDV: End-diastolic volume
EF: Ejection fraction
ES: End-systole
ESV: End-systolic volume
LV: Left ventricle/left ventricular
LVM: Left ventricular mass
MESA: Multiethnic Study of Atherosclerosis
MI: Myocardial infarction
MVR: Mass to volume ratio
PCA: Principal component analysis
PR: Precision recall
ROC: Receiver operating characteristic
RV: Right ventricle/right ventricular
SV: Stroke volume
TAPSE: Tricuspid annular plane systolic excursion
