# Supplementary figures and images for: Right ventricular shape and function: cardiovascular magnetic resonance reference morphology and biventricular risk factor morphometrics in UK Biobank
Source: J Cardiovasc Magn Reson. 2019 Jul 18;21:41. doi: 10.1186/s12968-019-0551-6 (PMC6637624; doi:10.1186/s12968-019-0551-6)

## Slide 1
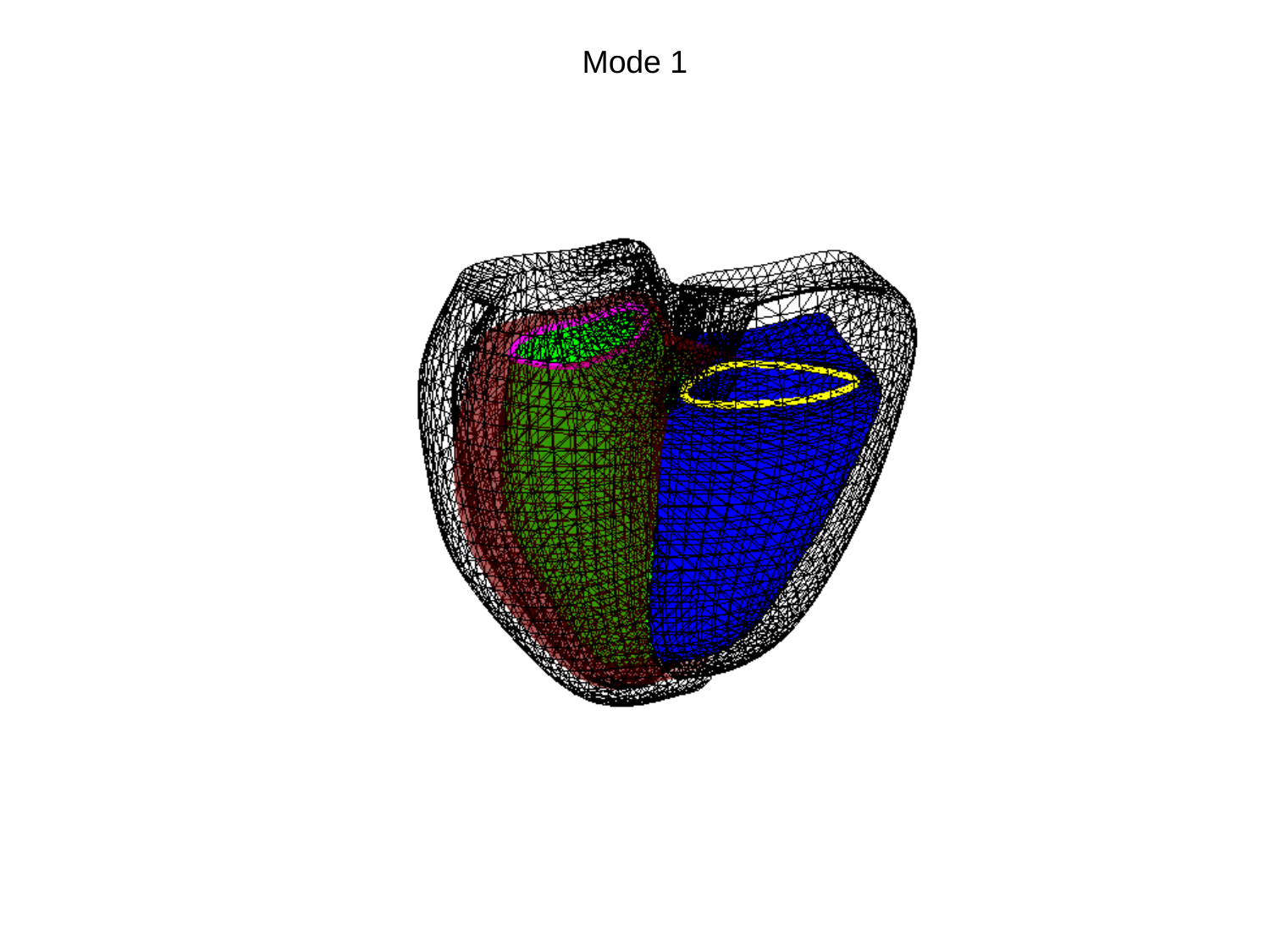

Mode 1

## Slide 2
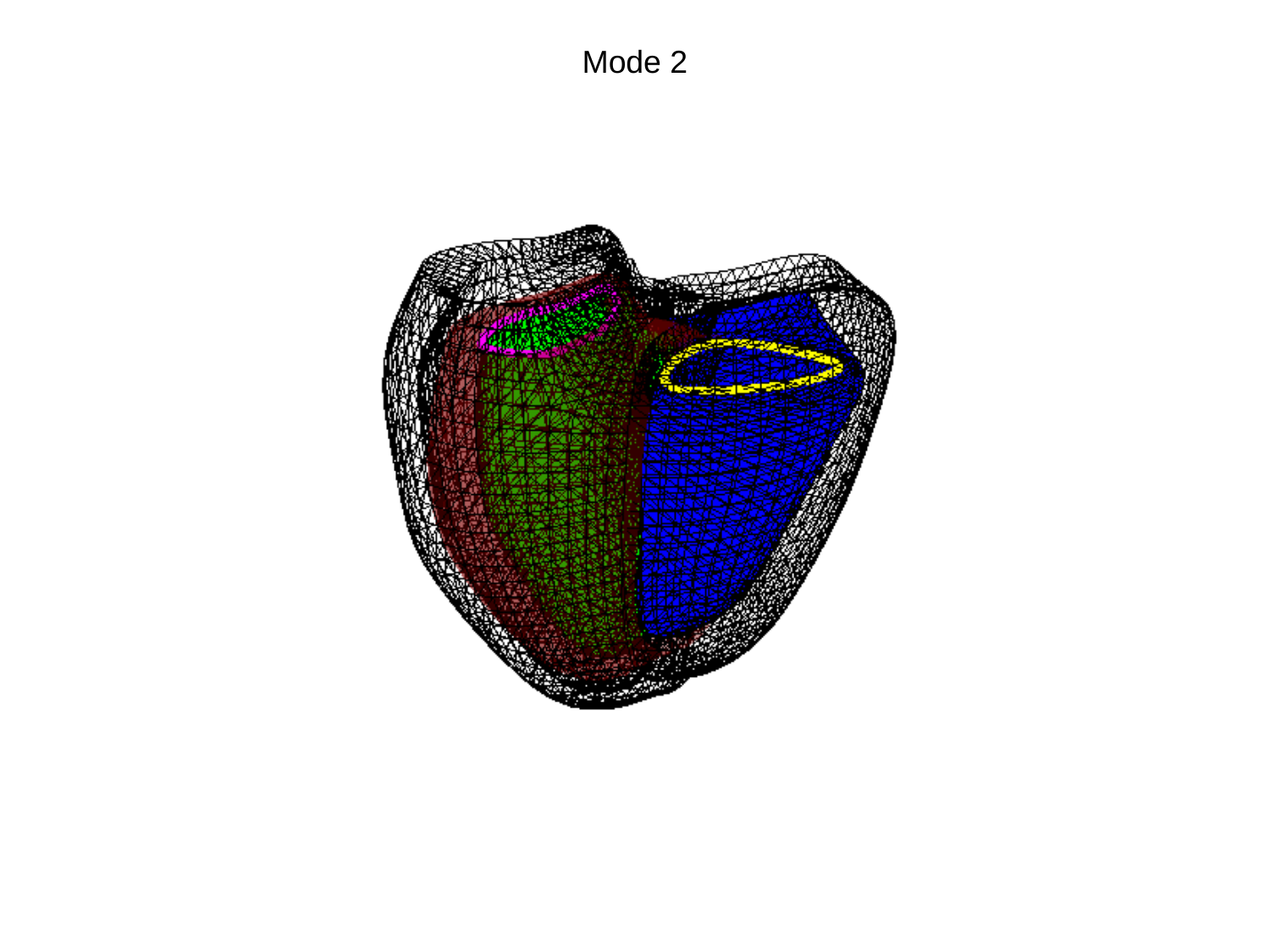

Mode 2

## Slide 3
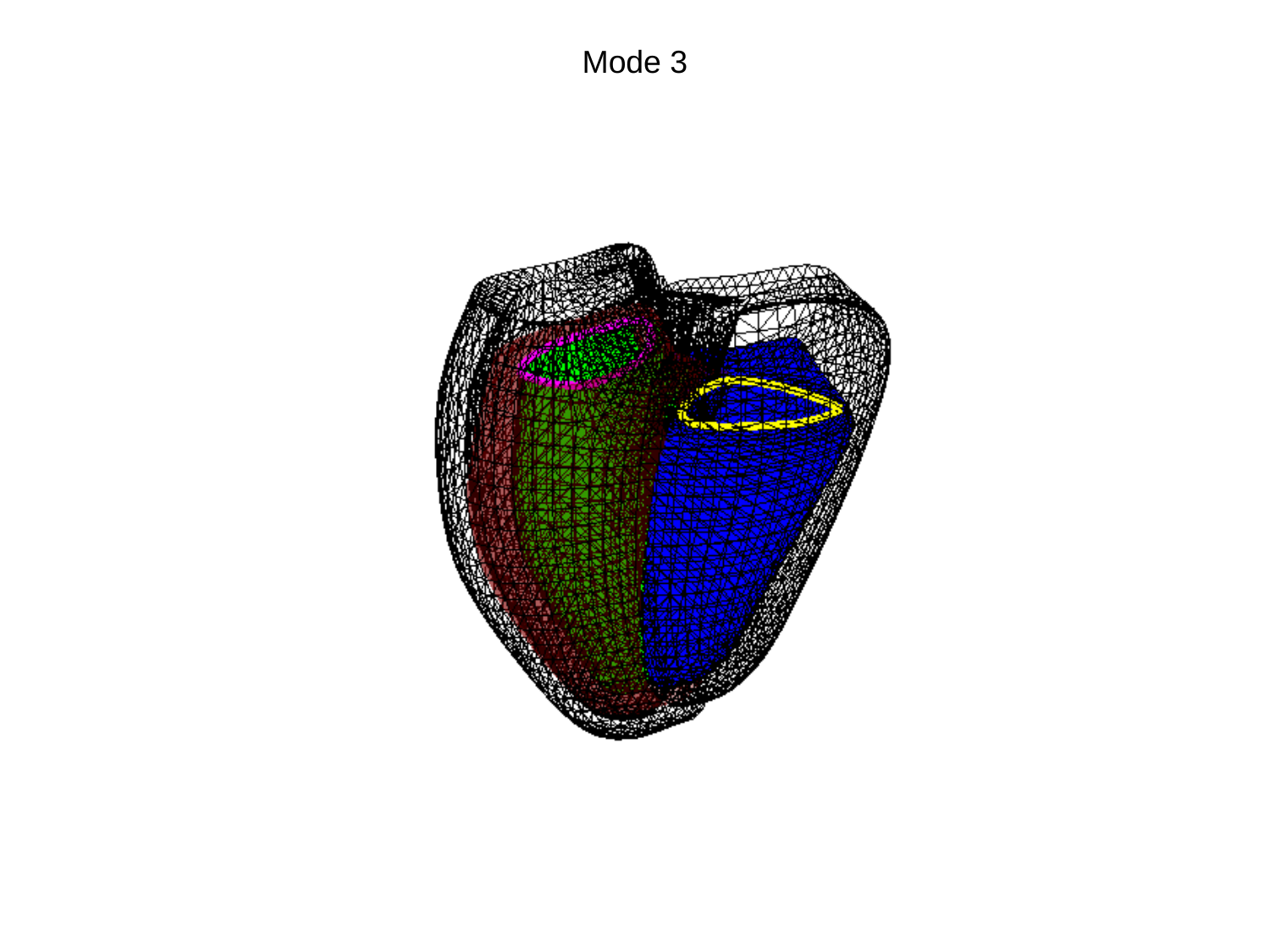

Mode 3

## Slide 4
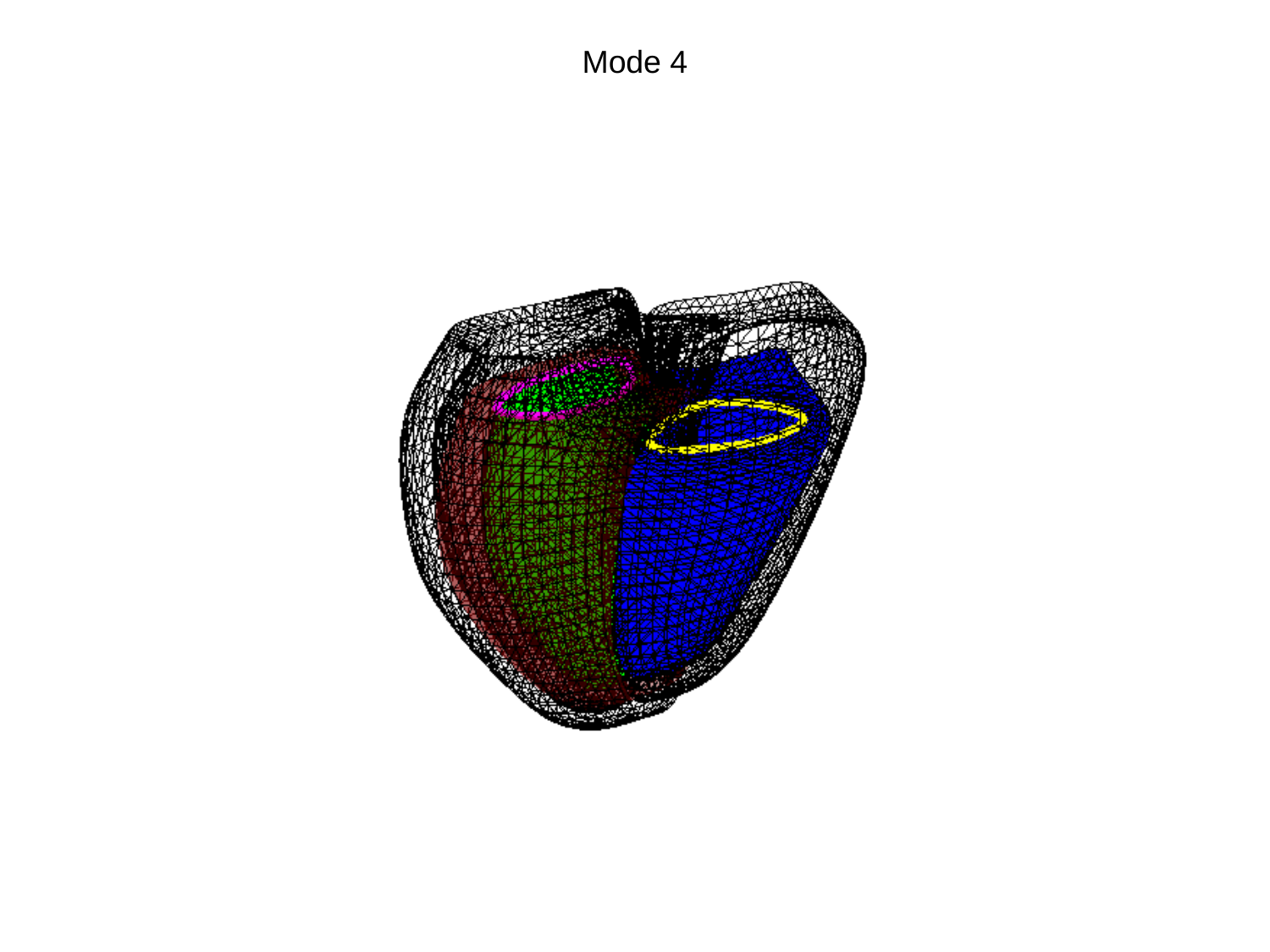

Mode 4

Supplement: Supplementary file 1 — Figure S2. Animations of the first four PCA components. (PPTX 2780 kb) [file 12968_2019_551_MOESM1_ESM.pptx]
