# Supplementary material for: Right ventricular shape and function: cardiovascular magnetic resonance reference morphology and biventricular risk factor morphometrics in UK Biobank
Source: J Cardiovasc Magn Reson. 2019 Jul 18;21:41. doi: 10.1186/s12968-019-0551-6 (PMC6637624; doi:10.1186/s12968-019-0551-6)
Supplement: Supplementary file 2 — Figure S1. Shape changes due to (a) previous MI, (b) angina, (c) obesity, (d) diabetes, (e) high cholesterol, (f) sex and (h) age, adjusted for other factors. Colors denote difference from the mean shape in mm (red outward, blue inward) for factor positive (Positive) and factor negative (Negative) groups. Outward directions are shown on the top right picture. Top row: anterior view of the RV (left) and LV (right). Bottom row: posterior view (left: LV, right: RV). Histograms show morphometric scores for reference healthy group and factor positive group. (PPTX 8496 kb) [file 12968_2019_551_MOESM2_ESM.pptx]

## Slide 1
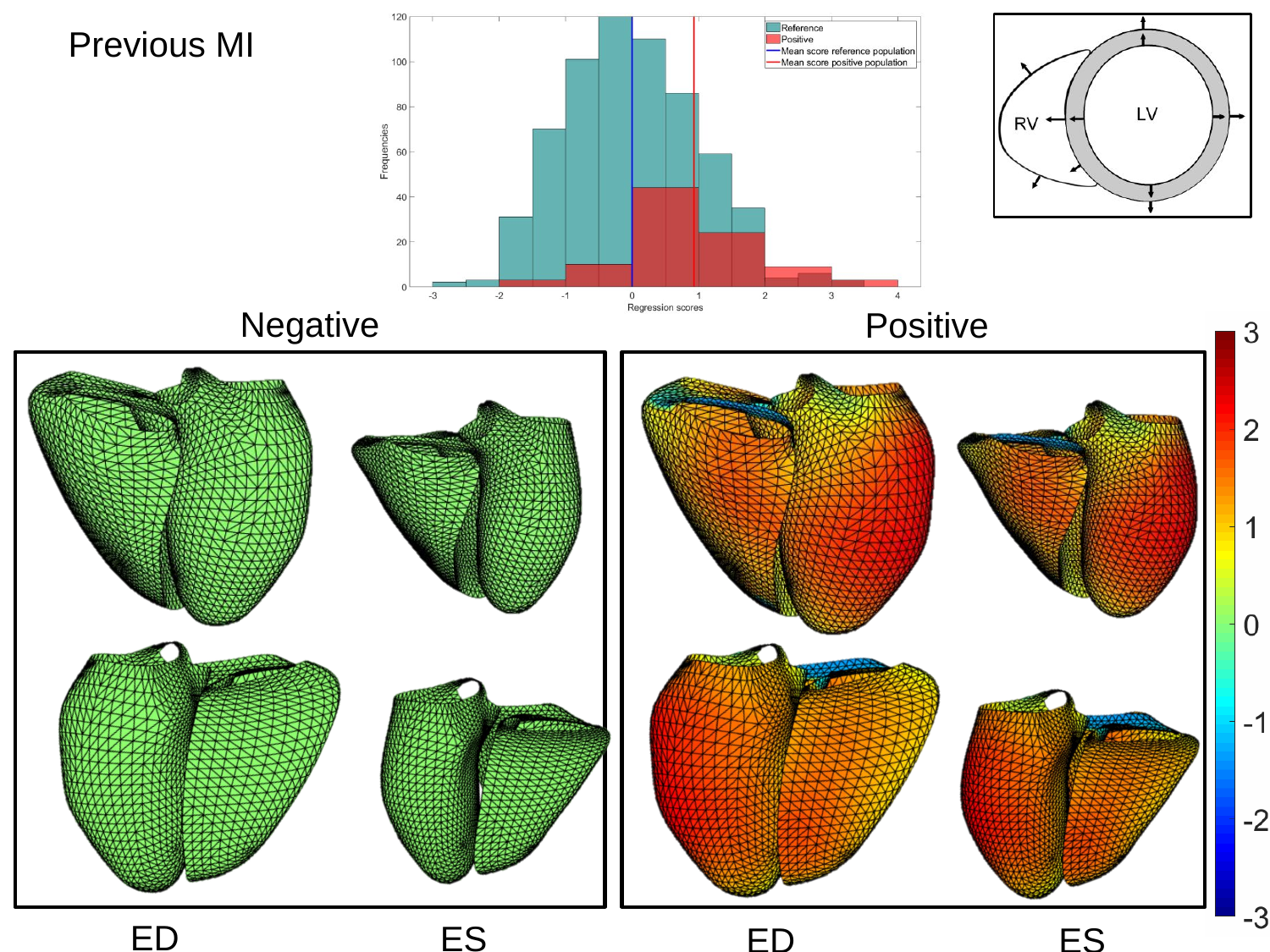

Previous MI
Negative
Positive
ED
ES
ED
ES

## Slide 2
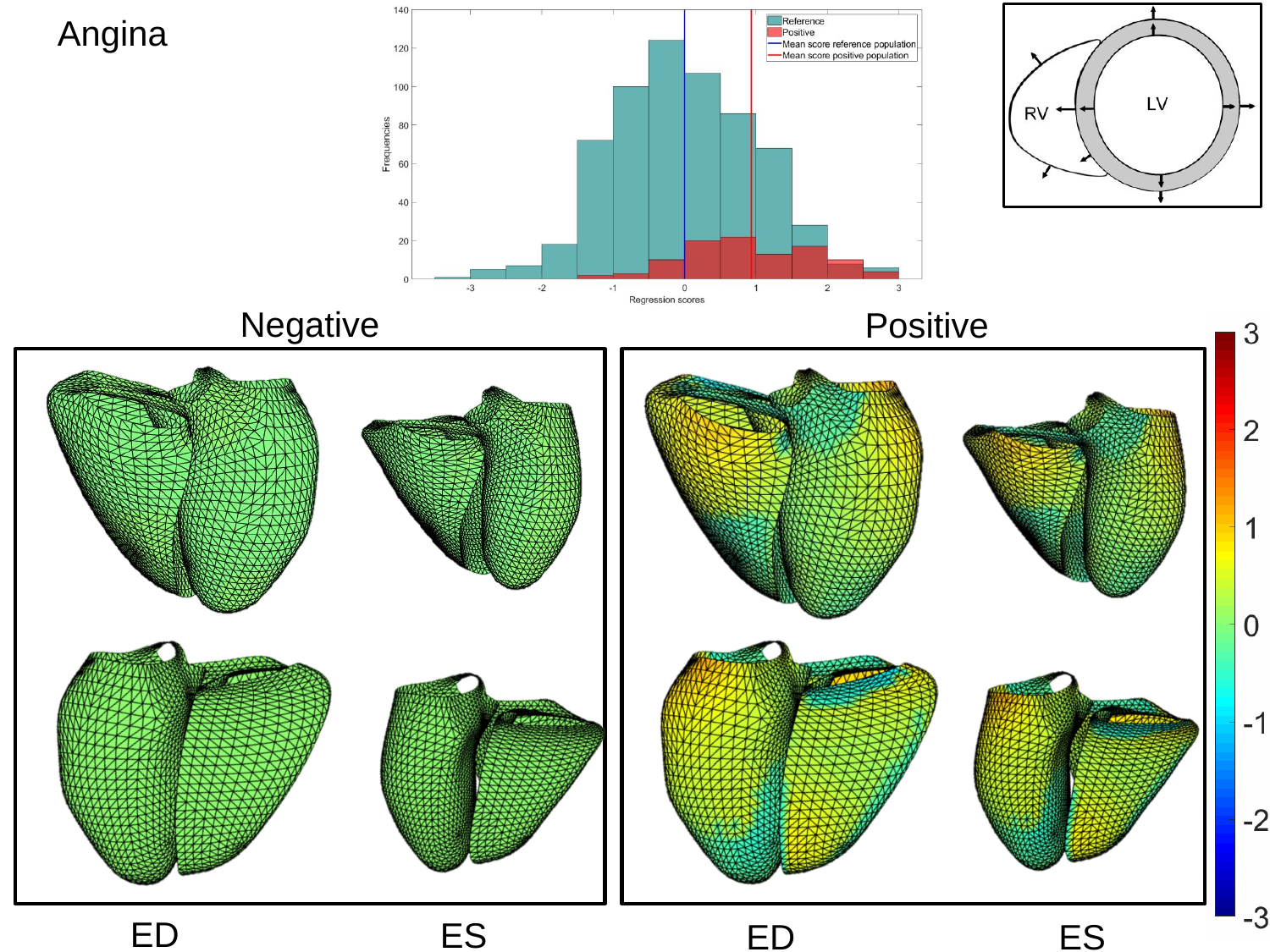

Angina
Negative
Positive
ED
ES
ED
ES

## Slide 3
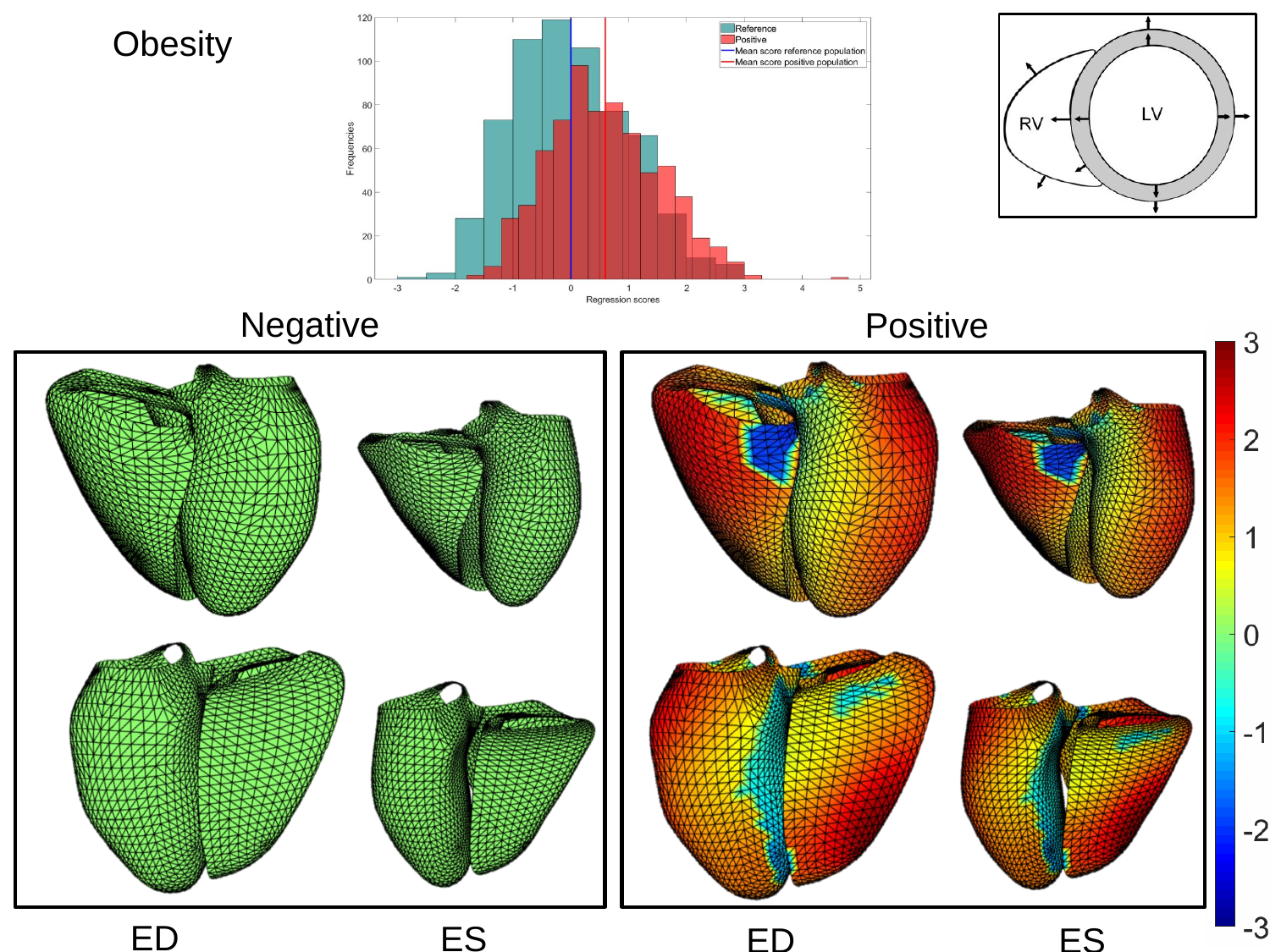

Obesity
Negative
Positive
ED
ES
ED
ES

## Slide 4
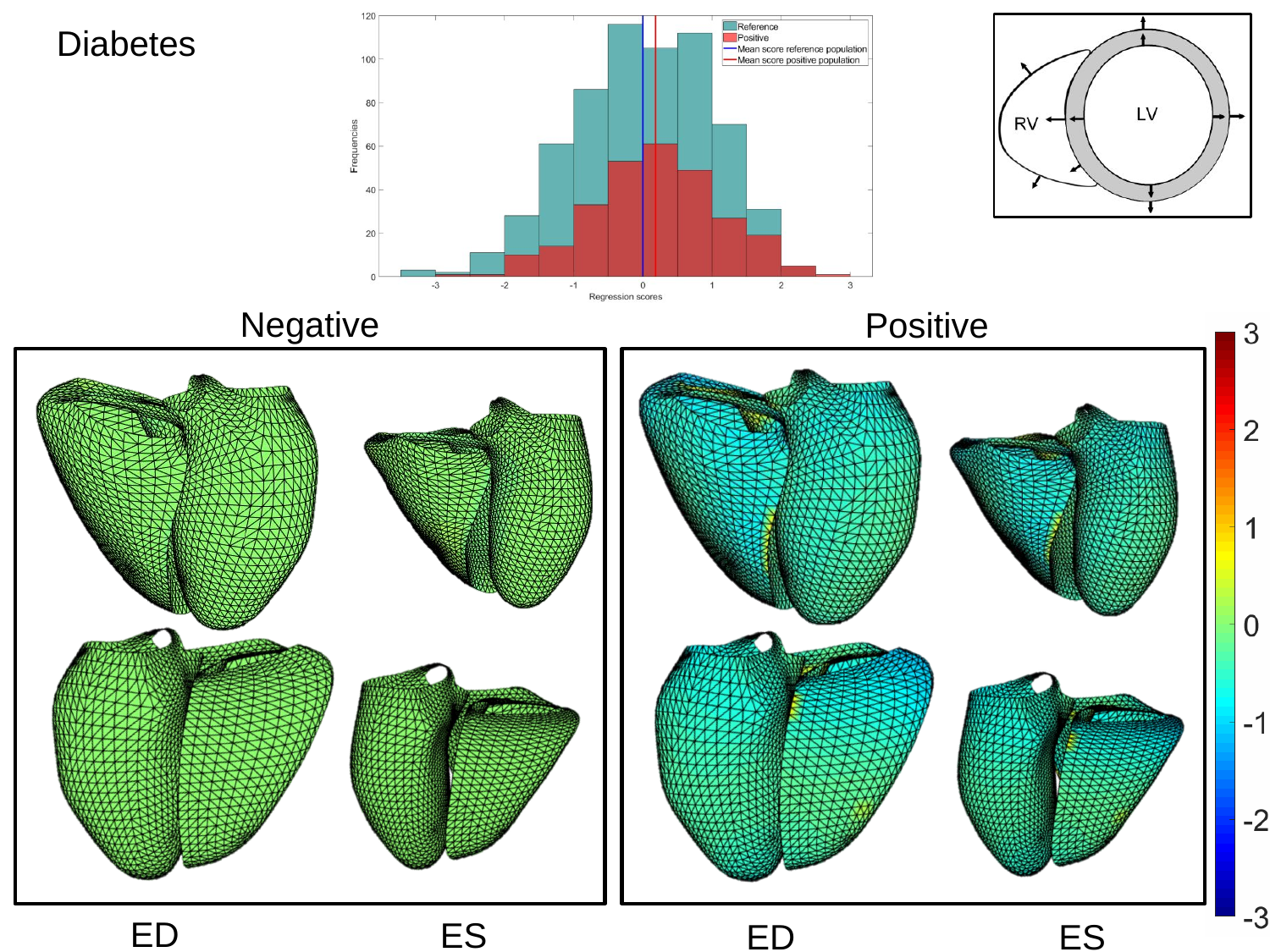

Diabetes
Negative
Positive
ED
ES
ED
ES

## Slide 5
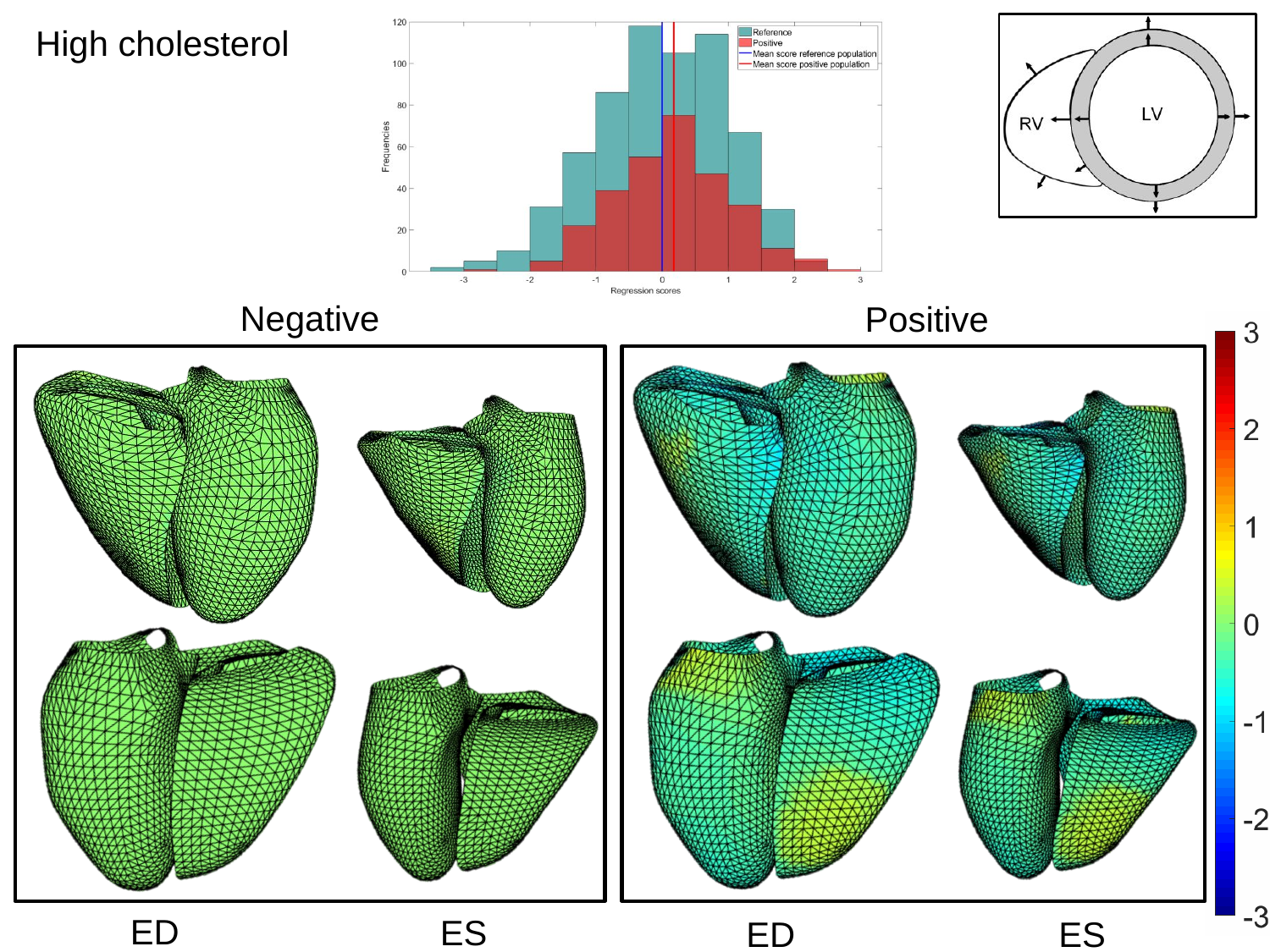

High cholesterol
Negative
Positive
ED
ES
ED
ES

## Slide 6
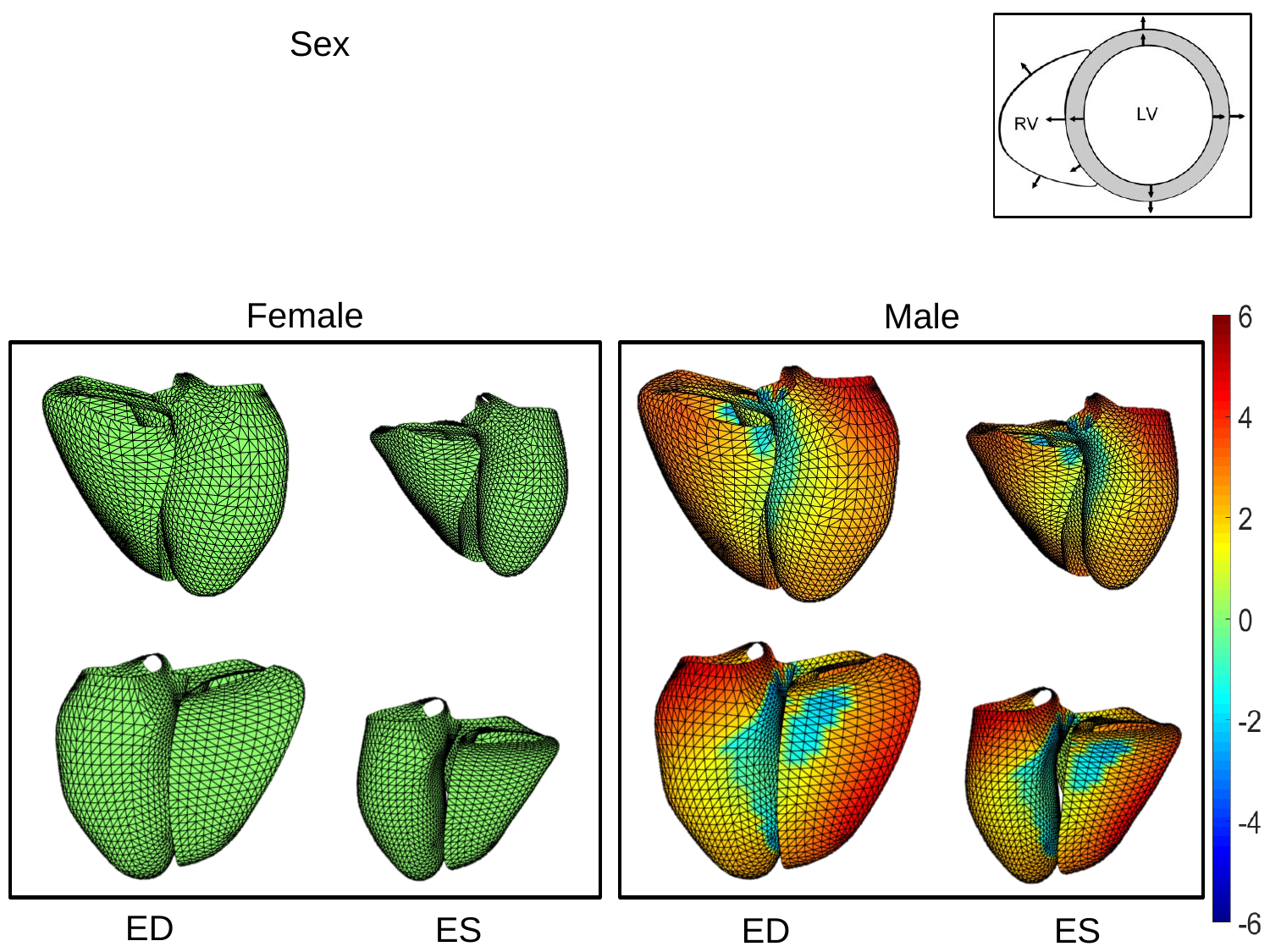

Sex
Female
Male
ED
ES
ED
ES

## Slide 7
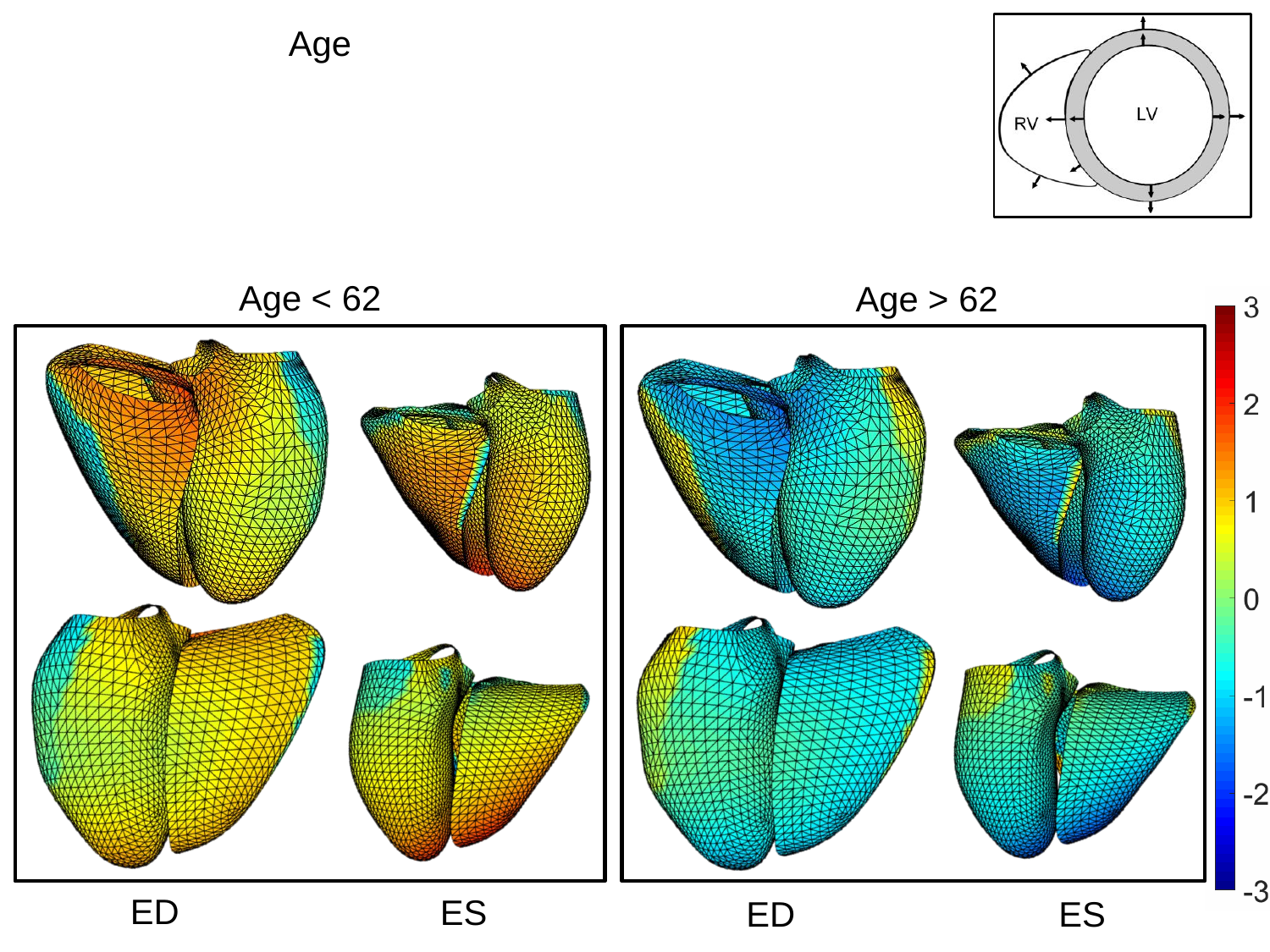

Age
Age < 62
Age > 62
ED
ES
ED
ES

## Slide 8
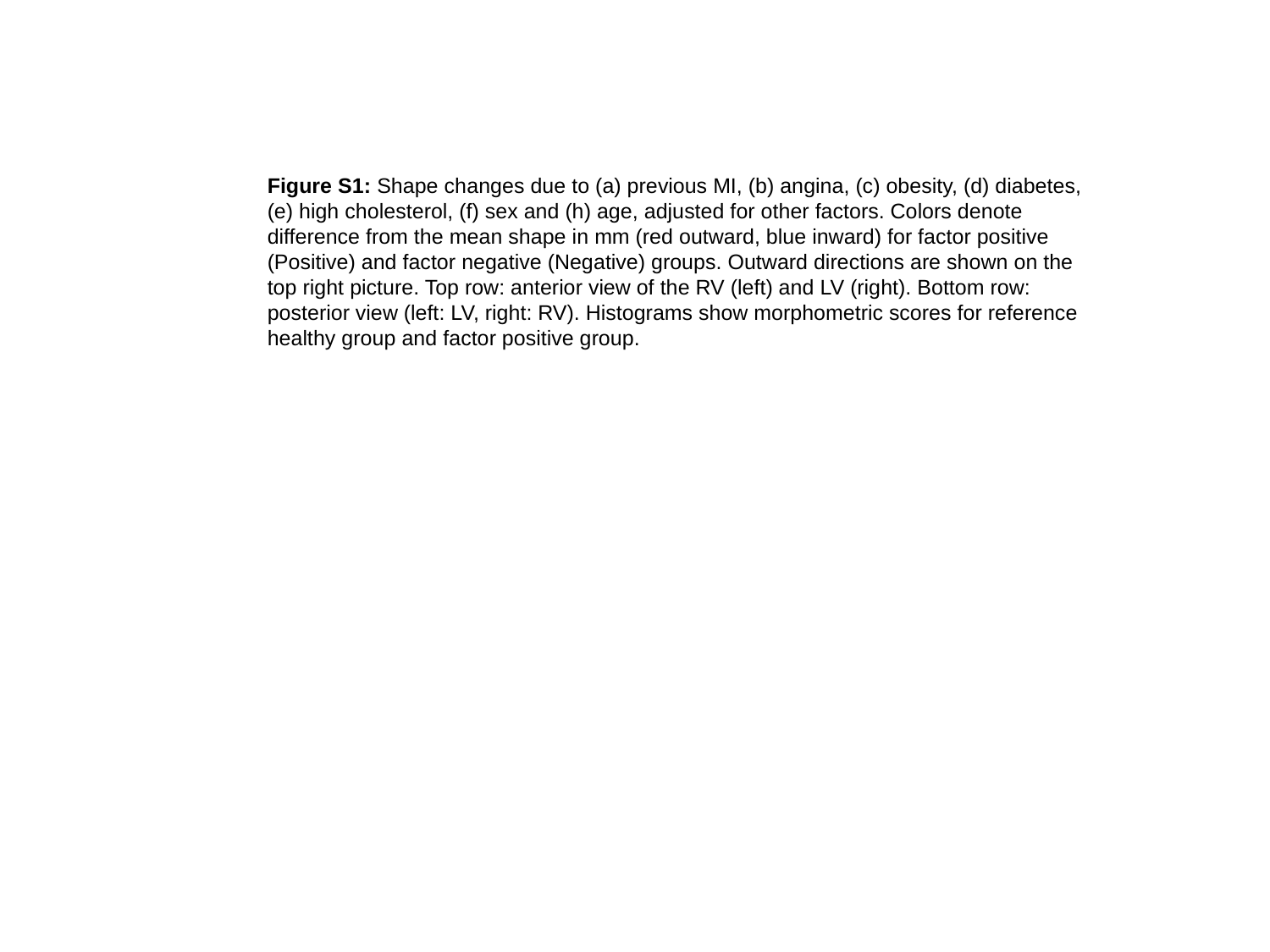

Figure S1: Shape changes due to (a) previous MI, (b) angina, (c) obesity, (d) diabetes, (e) high cholesterol, (f) sex and (h) age, adjusted for other factors. Colors denote difference from the mean shape in mm (red outward, blue inward) for factor positive (Positive) and factor negative (Negative) groups. Outward directions are shown on the top right picture. Top row: anterior view of the RV (left) and LV (right). Bottom row: posterior view (left: LV, right: RV). Histograms show morphometric scores for reference healthy group and factor positive group.
